# Effects of Akebia and Dandelion on Growth Performance, Inflammation, and Gut Microbiota in Weaned Rabbits

**DOI:** 10.3390/vetsci12111048

**Published:** 2025-11-01

**Authors:** Yawang Sun, Yan Zhang, Mussa Suleiman Mgeni, Xiaoyu Jiang, Yu Chen, Junqiu Zhang, Jingzhi Lv

**Affiliations:** 1 College of Animal Science and Technology, Southwest University, Chongqing 400716, China; 2 Department of Animal Science and Aquaculture, School of Agriculture, State University of Zanzibar (SUZA), Zanzibar P.O. Box 146, Tanzania

**Keywords:** weaned rabbits, plant extracts, growth performance, inflammatory factors, gut microbes

## Abstract

**Simple Summary:**

This study found that dandelion extract addition increased serum immunoglobulin levels and decreased the expression of inflammatory factors, and akebia extract addition decreased the mRNA expression of inflammatory factors and chemokines, contributing to the inhibition of inflammatory diseases. Moreover, akebia extract plays its anti-inflammatory function by creating an increased hind–gut microbiota diversity and increased abundance of certain microbiota species. The study indicated that dandelion extract and akebia extract have significant application value in improving the intestinal health and reducing the inflammatory response of weaned rabbits.

**Abstract:**

This study aimed to evaluate the individual and combined effects of dandelion extract and akebia extract on growth, inflammation, and gut microbiota in weaned rabbits. Using a two-factor randomized design, 120 rabbits (1.219 kg ± 0.077 kg, 35 days of age) were divided into four groups (10 replicates/group). Growth metrics (feed intake, fecal score, weight gain) and biological samples (blood, liver, jejunal/ileal mucosa, digesta) were analyzed over 28 days. Key results indicated that combined dandelion and akebia supplementation significantly increased daily weight gain during the first week (*p* < 0.05). Dandelion alone reduced liver Immunoglobulin G (IgG) and jejunal interleukin 6 (IL-6) (*p* < 0.05). Akebia supplementation decreased serum immunoglobulin A (IgA)/IL-6, liver interleukin 1β (IL-1β)/IL-6/IgG, and jejunal IL-1β/IL-6/IgG/IgA (*p* < 0.05). Gene expression revealed dandelion downregulated liver interferon-γ (IFN-γ)/interleukin 10 (IL-10) and jejunal IL-1β/interleukin 10 (TNF-α) (*p* < 0.05), while akebia suppressed hepatic C-C Motif Chemokine Ligand 3 (CCL3)/C-X-C Motif Chemokine Ligand 10 (CXCL10)/IFN-γ/IL-1β and mucosal IL-1β/IFN-γ (*p* < 0.05). A significant interaction effect (*p* < 0.05) between dandelion and akebia reduced ileal IL-6/IL-10/TNF-α and jejunal CXCL10/IL-10 mRNA expression. Akebia also increased cecal microbial diversity and the abundance of Oxalibacilli and Sutterella while reducing Firmicutes abundance (*p* < 0.05). In conclusion, both extracts modulated immunity and attenuated inflammatory responses through modulation of immunoglobulins, cytokines, and chemokines. Their combination demonstrated synergistic anti-inflammatory effects, alongside beneficial shifts in gut microbiota composition.

## 1. Introduction

As an important livestock sector, rabbit farming provides a valuable source of meat and fur worldwide, with China being one of the major producers. Therefore, developing effective strategies to enhance the health and productivity of weaned rabbits is of significant economic importance. The digestive system of weaned rabbits is still not well developed and is susceptible to indigestion, diarrhea, or disturbances in the structure of the gut microbes caused by improper feeding management or pathogenic microbial infestation, which could further trigger pathological reactions or even death [1]. Therefore, the improvement of weaned rabbits’ intestinal health and the prevention of diarrhea have long been regarded as an important issue for the development of rabbit farming [2]. Traditional treatments of diarrhea are mostly growth-promoting additives and antibiotic drugs. However, massive supplements of antibiotics induce drug residues of animal origins, environmental pollution, and increased drug resistance, and other problems are becoming increasingly serious [3]. Dandelion (Taraxacum Mongolicum), a perennial herb of the Asteraceae family, is native to Europe and commonly found throughout the southwestern and northwestern regions of China. Its ethanol extract mainly contains phenolic acid, flavonoid, and polysaccharide [4]. Dandelion polysaccharide extract can resist pathogenic bacteria and improve serum immunoglobulin M (IgM), immunoglobulin G (IgG) levels, and physical immunity in chickens [5], and relieve intestinal inflammation damage in weaned pigs [6]. Flavonoids can increase the amount of antioxidant active substance, dismutase (SOD), to resist oxidation. However, restrained by the organism’s selective metabolic absorption, the effective component in the dandelion is rarely distributed in the intestinal tract after absorption, making it less biologically active.

However, a theory called “Yinjing” [7] provided us with an idea of combining another guiding drug with dandelion. In traditional Chinese medicine, the theory of “Yinjing” is an important part of the theory of the nature of Chinese medicine. There are two meanings of this theory: one is that the drug itself can be concentrated in the organs and meridians to which it belongs, and the other is that the drug is directed to the appropriate part of the body. Modern medical research has found that the guiding drug probably affects the relative transfer protein and changes the structure of the cell membrane and cellular pH value to advance the permeability of the cell membrane and thus increases the concentration of medicine in the targeted organs or tissues [8]. Akebia (Akebia Quinata), which belongs to the genus Akebia within the family Lardizabalaceae, is distributed throughout the provinces and regions of China’s Yangtze River basin, as well as in Korea and Japan. It belongs to the small intestine channel and has the medical function of inflammation and oxidation resistance [9]. The β-sitosterol extracted from akebia can decrease the secretion of macrophage inflammatory factor interleukin 6 (IL-6) and tumor necrosis factor-α (TNF-α), and increase the activity of inflammatory factor interleukin 10 (IL-10) [10]. Oleanolic Acid extract can reduce the concentration of mouse liver interleukin 1β(IL-1β) [11]. Vanillic acid extract can reduce rat serum IL-1β, IL-6, and TNF-α levels to resist inflammation [12]. Studies have found that there was a close relationship between gut microbes and intestinal diseases, and intestinal microbiota is probably intimately connected with the physiological and biochemical functions of the body through regulating lymphocyte differentiation and inflammatory factor secretion [13,14]. Recent studies have pointed out the significant influence of intestinal microbiota on gut immunity through metabolism. In fecal samples from patients with inflammatory bowel disease, there is an increase in Aspergillus and Actinobacillus, and a decrease in the number of Firmicutes, which impairs the intestinal immune barrier [15]. Researchers have found that modulation of the intestinal microbiota of piglets can induce both short-term and long-term effects on their intestinal health [1,4]. Wen et al. [16] found that Baicalin can moderate the intestinal micro-environmental balance. According to Sun et al. [17], fermented Yu-Ping-Feng-San polysaccharides can maintain the intestinal barrier function and enhance physical immunity. Based on the channel tropism theory and the medicinal properties of dandelion and akebia, it is hypothesized that akebia, as a small intestinal tract-directing agent, may enhance the targeted delivery and strengthen the active compounds from dandelion extract, thereby potentiating its efficacy in improving intestinal health. We further propose that supplementation with a combination of dandelion and akebia will enhance systemic immunity while reducing pro-inflammatory cytokine expression and modulating gut microbiota composition in weaned rabbits. However, the effects of dandelion and akebia extracts, both individually and in combination, on gut health in weaned rabbits remain unclear. Therefore, the objective of this study is to evaluate the effects of individual and combined supplementation of dandelion and akebia extracts on growth performance, inflammatory status, and gut microbial structure in weaned rabbits.

## 2. Materials and Methods

### 2.1. Ethics Statement

The animal feeding and treatment were performed in accordance with relevant requirements. Ethical approval for the experiment was obtained from the Animal Ethics Committee of Southwest University of China for animal welfare (Number: 3167130267).

### 2.2. Experimental Animals and Feed

A total of 120 healthy, weaned Hyla rabbits (1.219 kg ± 0.077 kg, 35 days of age) were randomly allotted into four groups under a two-factor experimental design. Each group consisted of 10 replications, with 3 rabbits in each replication. Each factor contains 2 treatment levels, 0% and 0.5% Dandelion(D) or Akebia(A) extract in the basic diet. The composition of the basic diet is shown in Table 1. The two extracts used for feeding in this experiment were purchased from Nanjing Daosifu Biotechnology Co., Ltd. (Nanjing, Jiangsu, China), in which the active ingredients of dandelion extract were flavonoids with a content of 10%, and the active ingredients of akebia extract were saponins with a content of 7%.

Before the entry of test rabbits, the rabbitry is thoroughly swept and disinfected with Neosporin disinfectant and a flame gun. Three rabbits as one replication were group-housed in pens (70 cm × 80 cm × 70 cm) with a leakage floor, and each pen was equipped with a separate feeder and nipple drinker. The experiment lasted for 5 weeks, including 1 week of a pre-study period. Rabbits were fed with a basic diet during the pre-feeding stage, with a gradually increased feed amount from 50 g/d to 100 g/d. To avoid diarrhea caused by excessive feed intake, the feed amount during the 4 weeks of the experiment was, respectively, 120, 130, 140, 150 g/d, with free water access. The extract powder was mixed proportionally into the ration for feeding. During the formal experiment, the feed leftovers, diarrhea rate were daily recorded, while body weight was measured weekly to calculate the average daily gain.

### 2.3. Sample Collection and Management

After the trial, one medium-condition weaned rabbit in each replication was chosen to take blood from the anterior vein and collected in vacuum tubes without anticoagulants, and centrifugate (4 °C, 1100× *g*, 10 min) to obtain serum and then stored at −80 °C for further research. The rabbits were then slaughtered for tissue collection and food sample collection. Five-centimeter segments from the middle of the jejunal and ileal were longitudinally incised to harvest mucosal scrapings, which were preserved in 2 mL sterile tubes. One cubic centimeter tissues from the middle part of the liver were separated and placed in 1.5 mL sterile centrifuge tubes. The contents from the middle part of the cecum were collected in 5 mL sterilized centrifuge tubes. After being frozen in liquid nitrogen, all centrifuge tubes are quickly transferred to a refrigerator at −80 °C for storage.

### 2.4. Cytokine Content Detection

The enzyme-linked immunosorbent assay kit (Mlbio Co., Ltd., Nanjing, Jiangsu, China) was used to measure IL-1β, IL-6, immunoglobulin A (IgA), and IgG concentration in serum, liver, and intestinal mucosa. The measurement was performed in strict adherence to the kit instructions. The assay was performed according to the procedure outlined in our previous study [18]. Specifically, samples were diluted 5 times and added to each cell wall, then filled with enzyme reagent except for the blank wells. After incubation, each cell walls were washed with washing solution 5 times and then administered color development. The absorbance (OD value) of each well at 450 nm was determined with an enzyme meter.

### 2.5. RNA Extraction, cDNA Inversion Rate, and Real-Time PCR

About 1 g of jejunal and ileal mucosal tissues were taken, and total RNA was extracted based on silica gel membrane purification technology and following the procedure mentioned in our previous study [18] (The kit was purchased from Nanjing Vazyme Biotechnology Co., Ltd., Nanjing, China). The sample was first lysed with buffer and then transferred to a gDNA filter column for centrifugation, and the filtrate was collected and mixed with absolute ethanol. The mixture was then transferred to Fast-Pure gDNA-Filter Column III for centrifugation, and the filtrate was discarded. Then buffer RW1 and buffer RW2 were added successively for centrifugation, and the filtrate was discarded. Finally, RNA was eluted by centrifugation with enzyme-free sterile water. RNA concentration and purity were determined, and the obtained RNA was stored in a −80 °C refrigerator for future use. Total RNA was reverse-transcribed to synthesize cDNA, following the instructions of reverse transcription (HiScriptP^®^ III All-in-one RT Super-Mix Perfect for qPCR, Nanjing Vazyme Biotech Co., Ltd., Nanjing, China), and the synthesized cDNA was stored in −20 °C refrigerator. The qPCR was demonstrated following the procedure of our previous study. The primer sequences of the detected genes shown in Table 2 were created using Primer Premier 5.0 software, synthesized by China Shenzhen Huada Gene Co., Ltd, Shenzhen, China. The detected genes were TNF-α, IL-1β, IL-6, IL-10, INF-γ, C-X-C Motif Chemokine Ligand 10 (CXCL10), and C-C Motif Chemokine Ligand 3 (CCL3). The relative expression of the targeted gene took Glyceraldehyde-3-phosphate dehydrogenase (GAPDH) as the internal reference gene, and the calculation of relative mRNA expression of T-bet and GATA-3mRNA was based on $2^{-△△Ct}$.

### 2.6. Illumina MiSeq Double-Tailed Sequencing

The raw offline data from high-throughput sequencing underwent preliminary quality control screening based on sequence quality. Samples failing to meet quality standards were subjected to retesting or supplementary sequencing. Quality-filtered sequences were demultiplexed into libraries and individual samples according to their index and barcode information, followed by removal of the barcode sequences. The prepared libraries were sequenced on the Illumina MiSeq platform. Subsequent data analysis was performed using the BMKCloud online platform (https://www.biocloud.net accessed on 29 August 2023). Species composition of the samples was characterized through steps including read splicing and filtering, clustering and denoising, as well as taxonomic annotation and abundance analysis.

### 2.7. Data Statistical Analysis

Experimental pictures were plotted using GraphPad Prism 8.0, and SPSS 23.0 software was used to conduct a two-factor analysis of variance to compare the Main effect (whether dandelion and Akebia extract are added to the diet) and its interaction. The results were compared using Duncan’s method for multiple comparisons and expressed as mean ± standard error of the mean (SEM). The difference is statistically significant with *p* < 0.05.

## 3. Results

### 3.1. Influence of Dandelion and Akebia Extract on Weaned Rabbits in Growth

As shown in Table 3, there was a significant interaction (*p* < 0.05) between the addition of dandelion and akebia extract to the diet on both daily weight gain and feed-to-gain ratio of the rabbit in the first week. While dandelion extract promoted daily weight gain and feed efficiency without the addition of Akebia extract, it inhibited daily weight gain and feed efficiency when Akebia extract was added (*p* < 0.05).

### 3.2. Influence of Dandelion and Akebia Extract Addition on IL-1β, IL-6, IgA, and IgG Concentration of Weaned Rabbits

**Figure 1:**
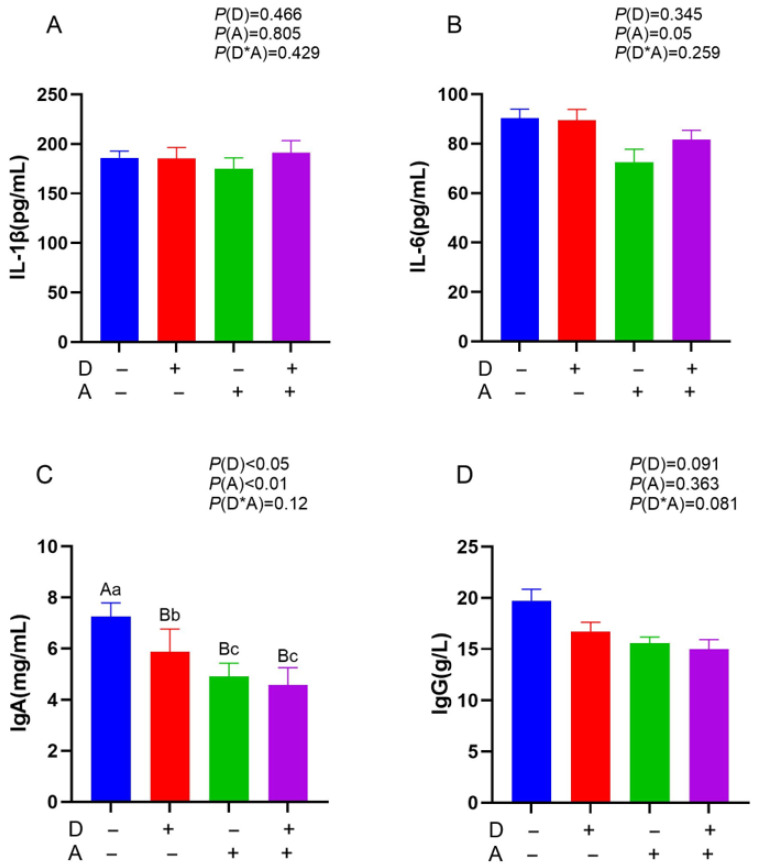
Influence of dandelion and akebia extract addition on serum IL-1β (**A**), IL-6 (**B**), IgA (**C**), and IgG (**D**) concentration of weaned rabbits. interleukin-1 beta (IL-1β); interleukin-6 (IL-6); immunoglobulin A (IgA); immunoglobulin G (IgG). Akebia extract (A), Dandelion extract (D). Different capital or lowercase letters indicate extremely significant difference (*p* < 0.01) or significant difference (*p* < 0.05) between treatments.

Figure 1 indicates a highly significant (*p* < 0.01) decrease in serum IgA concentration with the addition of akebia extract. In addition, there was a significant (*p* < 0.05) decrease in IgA with the addition of dandelion. There was no detectable interaction between the akebia and dandelion extracts in terms of IgA content, IL-6, IgG, along with IL-1β (*p* > 0.05).

**Figure 2:**
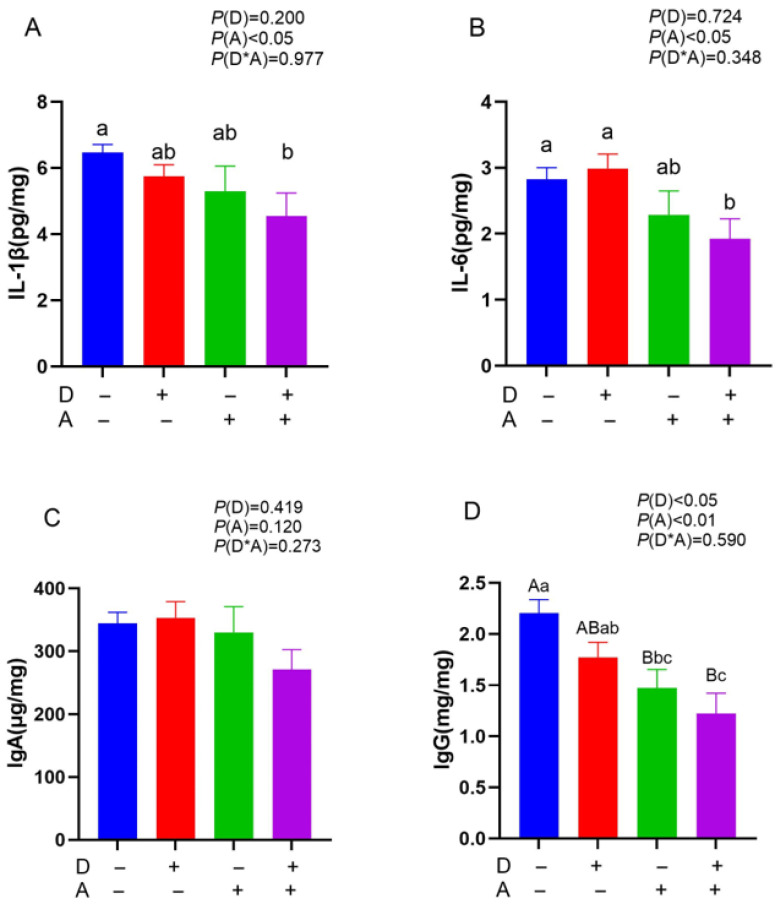
Influence of dandelion and akebia extract addition on liver IL-1β (**A**), IL-6 (**B**), IgA (**C**), and IgG (**D**) concentration of weaned rabbits. interleukin-1 beta (IL-1β); interleukin-6 (IL-6); immunoglobulin A (IgA); immunoglobulin G (IgG). A: akebia extract; D: dandelion extract. Different capital or lowercase letters indicate extremely significant difference (*p* < 0.01) or significant difference (*p* < 0.05) between treatments.

The addition of dandelion extract to the diet showed a significant (*p* < 0.05) decrease in the content of IgG in liver tissue (Figure 2). Concentration of IgG, IL-6, and IL-1β in liver tissue was significantly (*p* < 0.05) reduced with the addition of akebia extract to the diet. Significant interaction between dandelion and akebia was not found in the amounts of IL-1β, IL-6, IgA, and IgG in liver tissue (*p* > 0.05).

**Figure 3:**
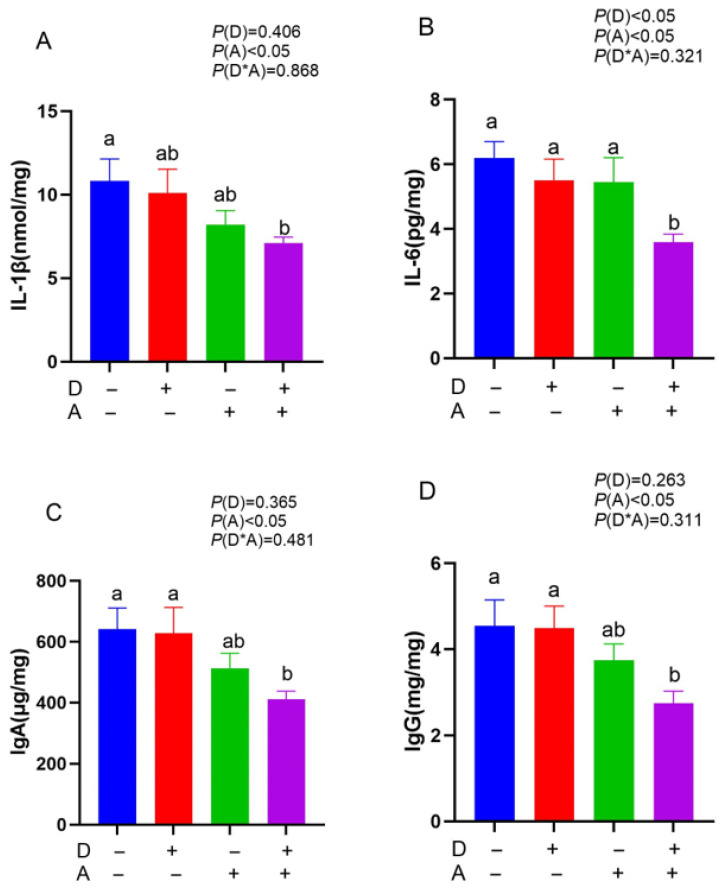
Influence of dandelion and akebia extract addition on jejunal mucosa IL-1β (**A**), IL-6 (**B**), IgA (**C**), and IgG (**D**) concentration of weaned rabbits. interleukin-1 beta (IL-1β); interleukin-6 (IL-6); immunoglobulin A (IgA); immunoglobulin G (IgG). A: akebia extract; D: dandelion extract. Different lowercase letters indicate a significant difference (*p* < 0.05) between treatments.

A significant (*p* < 0.05) decrease in the levels of IL-6 in the jejunal mucosa was observed following administration of dandelion extract, as shown in Figure 3. Additionally, after being exposed to akebia extract, the contents of IgA, IgG, IL-1β, and IL-6 in the jejunal mucosa were significantly (*p* < 0.05) decreased. There was no significant interaction between dandelion and akebia on cytokine and antibody content of the jejunal mucosa (*p* > 0.05).

**Figure 4:**
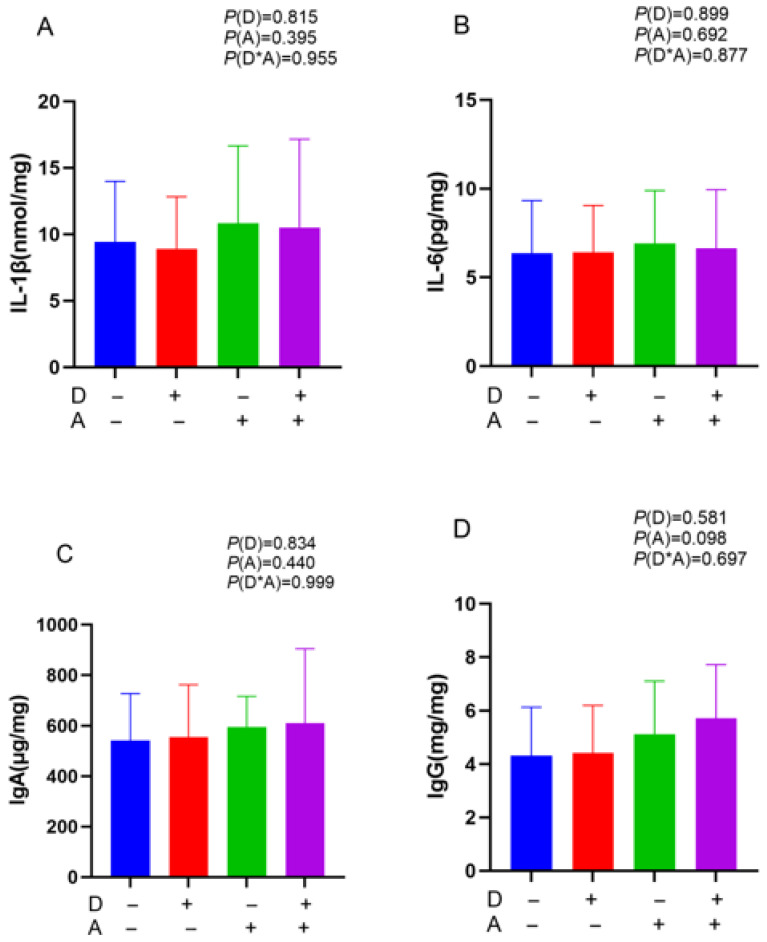
Influence of dandelion and akebia extract addition on ileal mucosa IL-1β (**A**), IL-6 (**B**), IgA (**C**), and IgG (**D**) concentration of weaned rabbits. interleukin-1 beta (IL-1β); interleukin-6 (IL-6); immunoglobulin A (IgA); immunoglobulin G (IgG). A: akebia extract; D: dandelion extract.

As shown in Figure 4, there was no significant influence of dandelion and akebia extracts on IgA, IgG, IL-1β, and IL-6 in the ileal mucosa (*p* > 0.05).

### 3.3. Influence of Dandelion and Akebia Extract on mRNA Expression Related to Genes of Inflammatory Response

**Figure 5:**
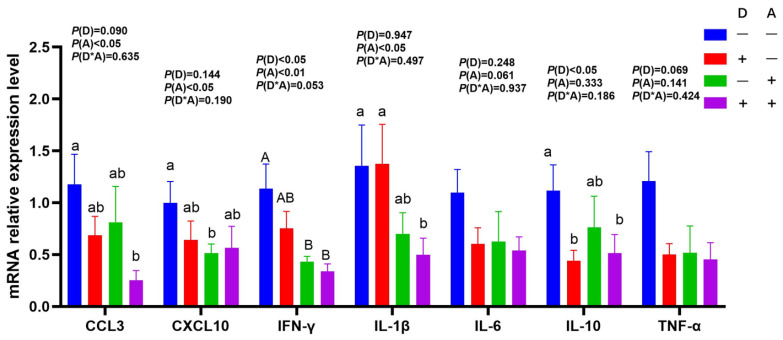
Influence of dandelion and akebia extract in liver mRNA expression related to genes of inflammatory response. Akebia extract (A); dandelion extract (D); interleukin-1 beta (IL-1β); interleukin-6 (IL-6); interleukin-10 (IL-10); tumor necrosis factor-alpha (TNF-α); interferon-gamma (IFN-γ); chemokine ligand 3(CCL3); chemokine (C-X-C motif) ligand 10 (CXCL10). Different capital or lowercase letters indicate extremely significant difference (*p* < 0.01) or significant difference (*p* < 0.05) between treatments.

As shown in Figure 5, in comparison to groups who received dandelion extract, it was shown that groups that were not treated with dandelion extract had significantly lower (*p* < 0.05) gene mRNA expression levels of IFN-γ and IL-10 in their liver tissue. Moreover, dandelion extract had a tendency to decrease the mRNA expression levels of TNF-αand CCL3 in liver tissue. The treatment of akebia extract in liver tissue led to a significant (*p* < 0.05) drop in the mRNA expression levels of IFN-γ, CCL3, CXCL10, and IL-1β.

**Figure 6:**
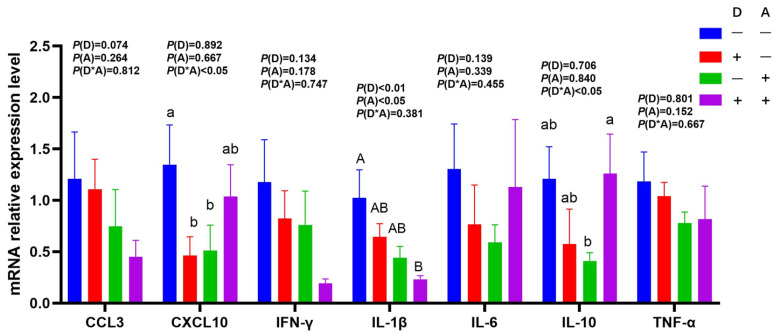
Influence of dandelion and akebia extract in jejunum mucosa mRNA expression related to genes of inflammatory response. Akebia extract (A); dandelion extract (D); interleukin-1 beta (IL-1β); interleukin-6 (IL-6); interleukin-10 (IL-10); tumor necrosis factor-alpha (TNF-α); interferon-gamma (IFN-γ); chemokine ligand 3(CCL3); chemokine (C-X-C motif) ligand 10 (CXCL10). Different capital or lowercase letters indicate extremely significant difference (*p* < 0.01) or significant difference (*p* < 0.05) between treatments.

The mRNA expression levels of IL-1β in the jejunum were significantly (*p* < 0.01) decreased following dandelion extract administration, as shown in Figure 6. In the jejunum, akebia extract significantly reduced IL-1β mRNA expression (*p* < 0.05). Significant interactions (*p* < 0.05) were seen in the mRNA expression levels of IL-10 and CXCL-10 between dandelion and akebia. Dandelion extract decreased IL-10 and CXCL10 mRNA expression without the addition of akebia extract, while the trend was reversed with the addition of akebia extract.

**Figure 7:**
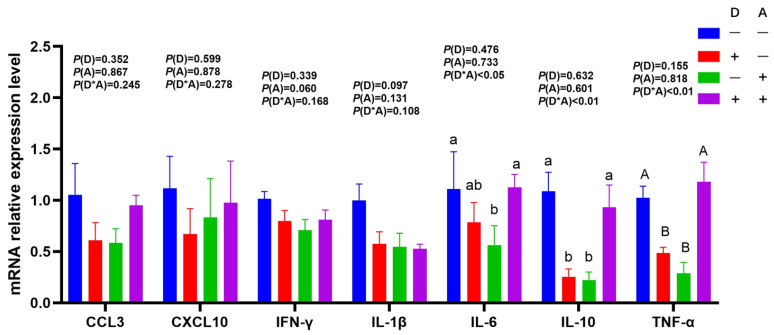
Influence of dandelion and akebia extract on ileum mucosa mRNA expression related to genes of inflammatory response. Akebia extract (A); dandelion extract (D); interleukin-1 beta (IL-1β); interleukin-6 (IL-6); interleukin-10 (IL-10); tumor necrosis factor-alpha (TNF-α); interferon-gamma (IFN-γ); chemokine ligand 3(CCL3); chemokine (C-X-C motif) ligand 10 (CXCL10). Different capital or lowercase letters indicate extremely significant difference (*p* < 0.01) or significant difference (*p* < 0.05) between treatments.

The mRNA expression levels of IL-10, IL-6, and TNF-α were found to exhibit a significant interaction (IL-6, *p* < 0.05; IL-10 and TNF-α, *p* < 0.01) between the extracts of dandelion and akebia (Figure 7). Dandelion extract decreased IL-10, IL-6, and TNF-α mRNA expression without the addition of akebia extract, while the trend was reversed with the addition of akebia extract.

### 3.4. Influence of Dandelion or Akebia Extract Addition on Weaned Rabbits’ Intestinal Microbial Flora

Compared with the control group, the group with akebia extract showed a significant rise (*p* < 0.05) in the Chao1 index, but a significant reduction (*p* < 0.05) in the Goods coverage index (Table 4). There was no significant interaction between dandelion and akebia extracts on weaned rabbits’ cecum flora’s alpha diversity index (*p* > 0.05).

### 3.5. Microbial Flora Composition of Samples

#### 3.5.1. Microbiota Composition of Weaned Rabbits in the Level of Phylum

**Figure 8:**
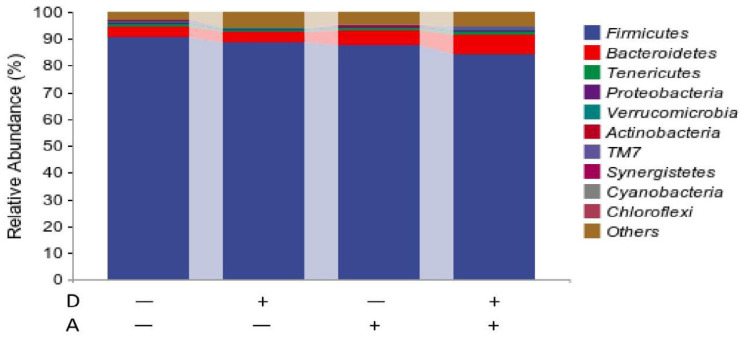
Microbiota composition of weaned rabbits at phylum level. Akebia extract (A), Dandelion extract (D), Standard error of mean (SEM). Symbol + or − stands for with 0% or 0.5% extract addition in the group.

The result at the phylum level is shown in Figure 8. The 10 species with the highest abundance at the phylum level were presented, and the rest species with lower abundance than the tenth phylum were classified into others with their relative abundance merged. The dominant phylum categories were Firmicutes, Bacteroidetes, Tenericutes, Verrucomicrobiota, Proteobacteria, and Actinobacteria. As can be seen from the stacked histogram of the percentage of each phylum, relative abundance greater than 1% is considered to be the dominant phylum. The dominant flora in the samples of the four groups was Firmicutes, with Bacteroidetes. The relative abundance of Tenericutes and Proteobacteria was less than 1% on average for each group.

As shown in Table 5, the addition of akebia extract to the diet significantly decreased (*p* < 0.05) the relative abundance of Firmicutes in the cecum of weaned rabbits. There was a trend of synergistic reduction in the relative abundance of Verrucomicrobiota by the extracts of akebia and dandelion. No significant interactions were found on the relative abundance of other phyla (*p* > 0.05).

#### 3.5.2. Microbiota Composition of Weaned Rabbits in the Level of Genus

**Figure 9:**
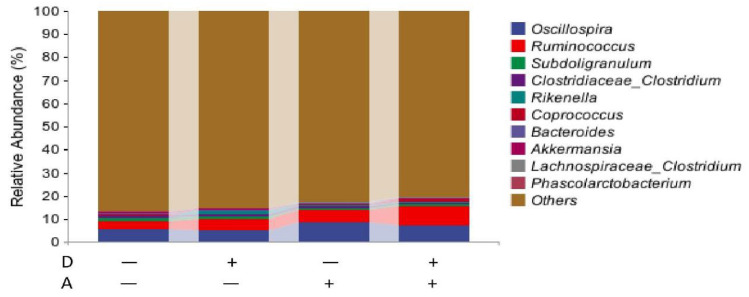
Microbiota composition of weaned rabbits at the level of genus. Akebia extract (A), Dandelion extract (D), Standard error of mean (SEM). Symbol + or − stands for with 0% or 0.5% extract addition in the group.

The results in the genus are presented in Figure 9. The top 20 genera were selected, and the rest were classified into others with merged relative abundance. The species with a relative abundance of more than 1% were regarded as dominant species. The dominant species in each group were Oscillospira and Ruminococcus, and the rare Subdoligranulum in the comparison groups was also classified as a dominating species.

As shown in Table 6, a significantly increased (*p* < 0.05) relative abundance of cecum Oxalobacter and Sutterella, as well as a significantly decreased (*p* < 0.05) relative abundance of Blautella and Lactobacillus, was observed with akebia extract addition. The relative abundance of Lactobacillus was significantly increased (*p* < 0.05) by dandelion extract. Dandelion and akebia extracts had a significant interaction effect (*p* < 0.05) on the relative abundance of Ruminococcus. Akebia extract can enrich the relative abundance of Ruminococcus without dandelion extract supplementation, while the trends were reversed with the addition of dandelion extract.

### 3.6. Species Differences and Marker Species Analysis

**Figure 10:**
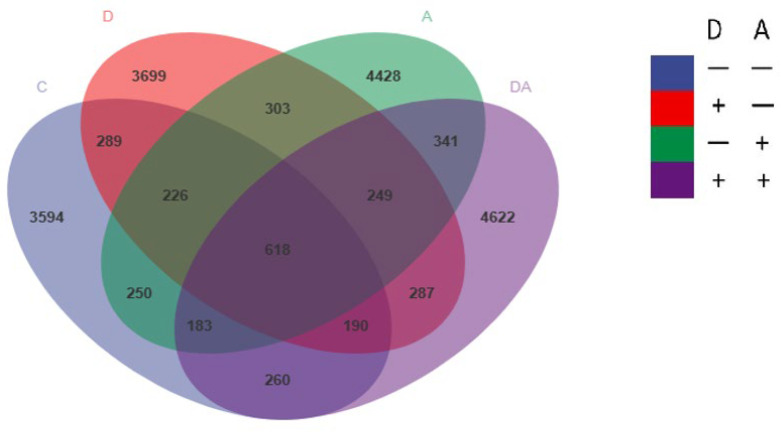
Effects of dandelion and akebia on cecum microbial Venn diagram of OTUs of weaned rabbits. Akebia extract (A); dandelion extract (D).

There were 19,539 OTUs in these four groups, of which 618 OTUs were found in all four groups. The unique OTUs in the control group were 3594, the unique OTUs in the supplementation of dandelion and supplementation of akebia group were 3699 and 4428, respectively, and 4622 unique OTUs were observed in the DA group (Figure 10).

**Figure 11:**
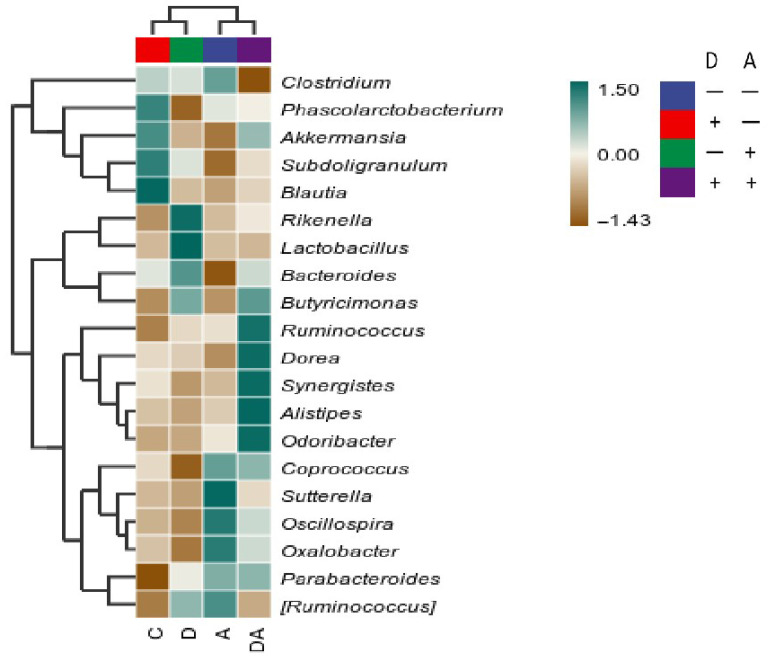
Differences in species abundance at the genus level of the top 20 genera in average abundance. Akebia extract (A); dandelion extract (D).

As shown in Figure 11, at the generic level, the relative abundances of Phascolarctobacterium and Coprococcus were found to be lower after dandelion extract was administered. Meanwhile, the abundance of Rikenella and Lactobacillus was found to be higher compared to other groups. In the same way, consumption of akebia extract was associated with higher relative abundances of Sutterella, Oscillospira, and Oxalobacter in the cecum as well as lower Bacteroides abundances. Furthermore, the relative abundances of Ruminococcus, Dorea, Synergistes, Alistipes, and Odoribacter increased in combination with the interactions between dandelion and akebia extract, whereas Clostridium’s abundance decreased. Furthermore, to assess the overall structural differences in microbial communities, beta diversity was evaluated, but the results suggest that the 24 rabbits in the four groups displayed no difference in gut microbial composition.

## 4. Discussion

Dandelion extract is rich in polysaccharide, which plays a vitally important role in the growth and development process of animals. The addition of dandelion and akebia extract significantly increased the daily gain of weaned rabbits corporately in the first week and significantly decreased the average feed efficiency of weaned rabbits in the first week. Our findings are consistent with a recent study in rabbits, which reported that dietary supplementation with dandelion significantly enhanced the growth performance and meat quality of weaned rabbits by modulating immune function and gut microbiota [19]. Dandelion extract promotes weight reduction and sustained normal body weight in high-fat diet rats [20], indicating the important role of dandelion in the growth of animals. According to Park et al. [21], after being treated with 200 mg/kg akebia, the mean body weight of mice was significantly increased, and the treatment group also showed better physical performance. The improvement in average daily gain in weaned rabbits supplemented with dandelion and akebia extracts can be attributed to their supportive role during the vulnerable post-weaning phase. At this stage, the digestive system of young rabbits is still immature and highly susceptible to dysfunction. The extracts helped enhance intestinal immune function, thereby promoting gut health. As a result, nutrient absorption and feed conversion ratio were improved, leading to a higher rate of weight gain per day.

The level of serum immunoglobulin is an important indicator of animal humoral immunity [22]. IgA is the dominant antibody in the exocrine fluid and plays its role in the local mucosal immunity, such as the respiratory tract and the intestinal tract. IgG is the dominant antibody produced by the organism after active immunity and is the main force of the anti-infection of the organism, playing immune activity of antibacterial, antiviral, and antitoxin activities, especially is important in humoral immunity [23]. The trend of decreased IgG level was observed with dandelion extract addition, indicating that dandelion and akebia extract modulated the immunity of weaned rabbits. This aligns with findings in weaned piglets, where dietary dandelion root extract was shown to alleviate systemic and intestinal inflammation, leading to improved gut health and growth performance [6]. Studies on oleanolic acid (a key constituent of akebia) have demonstrated its capacity to suppress the production of pro-inflammatory cytokines, including IL-6 and IL-1β, by inhibiting the NF-κB signaling pathway in murine models of liver inflammation [11,24].

Serum and intestinal tissue inflammatory cytokine levels reflect changes in intestinal inflammation [25]. The over-secretion of inflammatory mediators, including IL-1β, IL-6, and TNF-α, exhibits a potential pro-inflammatory effect [26]. Inhibiting the excessive release of these inflammatory mediators is crucial, as these specific cytokines play a pivotal role in the prevention or inhibition of various inflammatory diseases [27]. This experiment exhibited similar results to Choi et al. [10] and Wang et al. [11] that akebia extracts addition, significantly decreased serum IL-6 concentration and also significantly decreased IL-6 and IL-1β concentration in liver tissue and jejunum. The findings of this study are similar to those reported by Wang et al. [24], who demonstrated that the total flavonoid concentration in trifoliate akebia inhibits the expression of nuclear factor-kappa B (NF-κB) by reducing the levels of inflammatory factors such as TNF-α, IL-1β, and IL-6 in the liver. Similarly, our results suggest that akebia extract mitigates inflammation and protects the liver and intestines by decreasing the secretion of inflammatory factors, including IL-6 and IL-1β, in weaned rabbits. This protective effect against inflammation is consistent with the broader understanding of the role of anti-inflammatory agents in liver health.

The significant reduction in jejunal expression of interleukin (IL)-1β and tumor necrosis factor (TNF)-α following the addition of dandelion extract is consistent with the findings of Che et al. [28], who reported that dandelion significantly decreased the expression levels of TNF-α and IL-6 in mice with ulcerative colitis. Furthermore, our results align with those of Yao et al. [29], who demonstrated that taraxasterol inhibits inflammatory factors, including TNF-α, IL-10, and IL-6, by modulating the NF-κB signaling pathway, thereby attenuating inflammation. These findings collectively underscore the anti-inflammatory potential of dandelion extract and its active components in mitigating inflammatory responses in the gastrointestinal tract. Sang et al. [26] found that taraxasterol significantly inhibited the secretion of TNF-α, IL-6, IL-1β, IFN-γ, and IL-4 through regulating TLRs/NF-kB and Bax/Bc11-2 signaling pathway to prevent acute liver damage of mice. This experiment also indicated that dandelion extract addition significantly decreased the relative expression of liver IFN-γ, indicating that dandelion extract possibly prevents inflammation by decreasing the secretion of liver IFN-γ and jejunum TNF-α, IL-6, and IL-β of weaned rabbits.

A study on liver ischemia–reperfusion by Zhai et al. [30] indicated that IL-10 was an inflammatory factor that was produced from macrophages and could inhibit the secretion of inflammatory mediators such as TNF-α, IL-β, and IL-6 to prevent inflammation. Under normal physical conditions, pro-inflammatory and anti-inflammatory factors were dynamically balanced, while the over-inflammation triggered by numerous over-secreted pro-inflammatory factors increased the concentration of anti-inflammatory factor IL-10 [31]. The concentration of liver IL-10 was significantly decreased by dandelion extract addition, indicating that TNF-α and CCL3 had demonstrated a declining trend in weaned rabbits under a normal physical condition to maintain the dynamic balance of pro-inflammatory factors and anti-inflammatory, leading to the decreased concentration of IL-10. Macrophage M1 secretes both anti- and pro-inflammatory factors [32]. Pro-inflammatory factors, which could be divided into CC family (such as CCL3) and CXC family (such as CXCL10), could contribute to the migration and infiltration of inflammatory cells towards inflammatory spots and the secretion of pro-inflammatory factors such as IFN-γ, IL-6, and IL-8 [33]. Studies have found that chemokines such as CCL2, CCL3, and CXCL10 exhibited increased expression during the process of liver damage or liver inflammation [34,35], to which it was mentioned by Brass and Brenndörfer [36] that liver damage could be released by focusing on the recruitment and infiltration of neutrophils, decreased by chemokine receptors. Karthika et al. [37] and Zhou et al. [38] studied rectal cancer treatment through the compatibility of curcuma zedoary and 5-fluorouracil, finding that curcuma zedoary decreased the expression of mRNA and protein of liver and colon CCL3 and CXCL10. Wang et al. [24] found that pre-treatment with oleanolic acid in a mouse model of liver ischemia–reperfusion injury significantly reduced neutrophil recruitment and inflammation. The results of this experiment indicated that akebia with extract rich in oleanolic acid significantly decreased mRNA expression of liver CCL3 and CXCL10, implying that akebia plays its anti-inflammatory function through inhibiting chemokine CCL3 and CXCL10 secretion.

This experiment studied whether dandelion or akebia extract would affect the gut diversity of weaned rabbits by Illumina MiSeq technology, which made the detection data more comprehensively covered and the microbiota composition and variation verily and comprehensively reflected. Intestinal microbiota are closely related to the physical functions of the organism [39], and rabbits have a particularly developed cecum, making the whole digestive tract susceptible to various pathological reactions and causing digestion and absorption problems when the normal cecum flora is destroyed, which would even decrease organism immunity and lead to systemic pathological reaction [40]. The results of this experiment exhibited a significantly increased Chao1 index and an increased Shannon index, although not significantly, thus it can be implied that akebia extract increased species abundance and diversity in the cecum of weaned rabbits. The results of this experiment showed that Firmicutes and Bacteroidetes were the most abundant at the phylum level, and these two phyla accounted for over 90%, which is consistent with the study of Eckburg et al. [41]. Firmicutes were an important source of butyrate, which was the dominant cellular energy source. The decrease in Firmicutes might trigger or worsen the local inflammatory reaction [42]. Bacteroidetes is the flora involved in the metabolism of sugar, bile acid, and steroids, such as decomposing peptone or glucose and producing metabolites such as acetic acid, formic acid, lactic acid, and propionic acid, with its main function of absorbing and decomposing polysaccharides [43]. The decreased ratio of Firmicutes and Bacteroidetes directly affects the digestive tract flora for the metabolism of dietary fiber, leading to a decreased short-chain fatty acid (SCFA) concentration. SCFAs are an important product from the dietary fiber fermented by gut probiotics, mainly involving acetic acid, propionic acid, butyric acid, etc. SCFAs prevent inflammation through significantly declining pro-inflammatory chemotactic factors such as CCL2, CCL3, CXCL10, and CXCL11, so as to increase the anti-inflammatory factors such as IL-10. Akebia extract addition significantly decreased the abundance of Firmicutes and increased the abundance of Bacteroidetes, although the difference did not reach a significant level. The synergistic effect of Mucuna pruriens and Taraxacum officinale was more effective in regulating the structure of intestinal microbiota and increasing the abundance of beneficial flora than the effects of Mucuna pruriens and Taraxacum officinale alone.

Oscillospira and Ruminococcus both ferment fibers to produce SCFAs, which can prevent inflammation. The decreased Oscillospira abundance was observed in inflammatory diseases and was positively related to human health [44]. Through Beta diversity, it was found that the extracts of dandelion and akebia can significantly improve the diversity of the intestinal microbiota and increase the abundance of butyric acid-producing bacteria, such as Blautella. The results of this experiment implied addition of dandelion extract or akebia extract potentially exhibited the trend of increased Oscillospira and Ruminococcus abundance, indicating that dandelion or akebia extract prevents inflammation through increasing the abundance of Oscillospira and Ruminococcus.

## 5. Conclusions

Dietary supplementation with dandelion and akebia extracts synergistically improves weaned rabbit health by balancing immune function, reducing inflammation, and modulating cecal microbiota, offering viable natural alternatives in non-antibiotic rabbit production.

## Figures and Tables

**Table 1:** Composition and nutrient levels of the basic diet of the rabbit.

Ingredient	Content (%)	Nutrient Level	Content
Alfalfa powder	36	Digestible Energy/(MJ·kg^−1^)	12.02
Corn	25	Crude protein (%)	16.78
Soybean meal	12	Ether extract (%)	3.11
Wheat bran	23.5	Crude fiber (%)	12.92
Fish meal	1.5	Calcium (%)	0.79
CaHCO_3_	1	Phosphorus (%)	0.64
NaCl	0.5		
Premix ^1^	0.5		
Total	100		

^1^ The premix provided the following per kilogram of the diet: Fe 100 mg, Cu 15 mg, Zn 70 mg, Mn 15 mg, VA 1000 IU, VD3 1000 IU, VE 50 mg, niacin 50 mg, D-pantothenic acid 50 mg, choline 1000 mg.

**Table 2:** Primer sequence of fluorescent quantitative PCR.

Gene	Primer Sequence (5′–3′)	Product Fragment Length/bp	Gene Bank Number
CCL3	F:GCTCTCAGCACCCAGGTCTT	142	100348776
	R:GAGCACTGGCTGCTGGTCTC		
CXCL10	F:CCTCCAGTCTCAGCACCATGAATC	138	100353112
	R:GATGCAGGTACAGCGTACAGTTCTA		
IFN-γ	F:TTCCCAAGGATAGCAGTGGT	160	AB010386
	R:TGAAGCCAGAAGTCCTCAAAA		
IL-1β	F:GCTGGAGAGTGTAGAC	136	M26295
	R:CTGAGAGGTGCTGATG		
IL-6	F:TTCGGGGCTGATGGAGTT	83	AF169176
	R:TGCTGACCCTGGTGTTTTCTT		
IL-10	F:GCCCTGTGGGATTTGAGTGT	100	KT279661
	R:CAGGCACAGAAGGAATGGAA		
TNF-α	F:CTCCTACCCGAACAAGGTCA	138	M12845
	R:CGGTCACCCTTCTCCAACT		

**Table 3:** Influence of dandelion and akebia extract on weaned rabbits in growth.

ITEMS	A−	A−	A+	A+	SEM	*p*-Value		
	D−	D+	D−	D+		D	A	D*A
Body weight(kg)								
Initial	1.2	1.2	1.2	1.2	0.051	0.919	0.888	0.956
Week 1	1.4	1.4	1.5	1.4	0.045	0.613	0.377	0.337
Week 2	1.7	1.8	1.8	1.8	0.058	0.714	0.463	0.365
Week 3	2.0	2.1	2.1	2.1	0.102	0.863	0.763	0.687
Week 4	2.1	2.2	2.2	2.2	0.222	0.626	0.730	0.918
Average daily gain(g/d)								
Week 1	27.9 ^b^	32.0 ^a^	33.1 ^a^	30.5 ^ab^	2.287	0.527	0.136	0.009
Week 2	46.2	45.9	46.0	45.5	2.870	0.850	0.879	0.965
Week 3	44.0	42.7	42.1	42.7	5.312	0.945	0.848	0.839
Week 4	20.8	21.1	19.0	23.8	4.183	0.677	0.94	0.717
Average feed intake(g/d)								
Week 1	115.6	115.8	115.8	115.7	0.242	0.255	0.791	0.099
Week 2	129.0	130.0	129.4	130.0	1.197	0.196	0.784	0.784
Week 3	138.6	140.0	138.0	137.8	3.265	0.710	0.401	0.612
Week 4	147.8	148.0	147.9	147.9	0.162	0.415	0.900	0.280
Feed/gain ratio								
Week 1	4.2 ^a^	3.7 ^b^	3.6 ^b^	3.9 ^ab^	0.285	0.446	0.131	0.006
Week 2	2.9	2.9	2.8	2.9	0.230	0.950	0.897	0.785
Week 3	3.5	3.9	3.4	3.5	0.880	0.473	0.581	0.734
Week 4	7.0	7.0	7.3	6.2	1.348	0.522	0.291	0.534
Diarrhea rate	0.2	0.2	0.2	0.1	0.144	0.412	0.864	0.926

Akebia extract (A), Dandelion extract (D), Standard error of the mean (SEM). Symbol + or − stands for with 0% or 0.5% extract addition in the group. Different lowercase letters indicate a significant difference (*p* < 0.05) between treatments.

**Table 4:** Influence of dandelion and akebia extract on Alpha diversity of weaned rabbits.

Alpha Diversity Index	A−	A−	A+	A+	SEM	*p*-Value		
	D−	D+	D−	D+		D	A	D*A
Chao1	1258.502 ^b^	1345.605 ^ab^	1556.967 ^a^	1515.186 ^ab^	278.423	0.837	0.044	0.561
Observed species	1135.117	1190.867	1346.000	1334.033	242.477	0.826	0.086	0.734
Shannon	7.965	7.955	8.407	8.250	0.697	0.778	0.222	0.805
Simpson	0.981	0.976	0.988	0.985	0.016	0.580	0.225	0.880
Faith_pd	64.882	68.342	74.509	74.097	9.657	0.694	0.058	0.618
Pielou_e	0.786	0.779	0.809	0.796	0.050	0.641	0.357	0.862
Goods coverage	0.988 ^a^	0.984 ^ab^	0.980 ^b^	0.981 ^ab^	0.005	0.621	0.009	0.253

Akebia extract (A), Dandelion extract (D), Standard error of the mean (SEM). Symbol + or − stands for with 0% or 0.5% extract addition in the group. Different lowercase letters indicate a significant difference (*p* < 0.05) between treatments.

**Table 5:** Microbiota composition of weaned rabbits at phylum level.

Items %	A−	A−	A+	A+	SEM	*p*-Value		
	D−	D+	D−	D+		D	A	D*A
Firmicutes	83.345 ^a^	85.987 ^ab^	90.213 ^ab^	90.751 ^b^	5.257	0.547	0.037	0.690
Bacteroidetes	8.381	5.262	4.314	3.887	4.167	0.417	0.218	0.536
Tenericutes	0.651	0.712	0.493	0.733	0.470	0.552	0.787	0.725
Proteobacteria	0.613	0.547	0.444	0.557	0.227	0.849	0.516	0.468
Verrucomicrobiota	0.431	0.165	0.325	0.448	0.198	0.487	0.390	0.069
Actinobacteria	0.268	0.359	0.178	0.292	0.140	0.152	0.267	0.872
TM7	0.597	0.125	0.138	0.086	0.368	0.198	0.220	0.299
Synergistetes	0.208	0.074	0.032	0.071	0.088	0.439	0.160	0.172
Cyanobacteria	0.073	0.062	0.065	0.062	0.043	0.467	0.689	0.787
Chloroflexi	0.000	0.004	0.000	0.000	0.003	0.210	0.210	0.210
Others	5.459	6.737	3.818	3.13	2.915	0.839	0.086	0.507

Akebia extract (A), Dandelion extract (D), Standard error of the mean (SEM). Symbol + or − stands for with 0% or 0.5% extract addition in the group. Different lowercase letters indicate a significant difference (*p* < 0.05) between treatments.

**Table 6:** Microbiota composition of weaned rabbits at the level of genus.

Items %	A−	A−	A+	A+	SEM	*p*-Value		
	D−	D+	D−	D+		D	A	D*A
Oscillospira	5.798	5.141	8.740	6.739	3.336	0.436	0.190	0.692
Ruminococcus	3.427	5.115	5.236	6.551	1.762	0.105	0.081	0.835
Subdoligranulum	1.292	0.992	0.608	1.072	0.812	0.842	0.466	0.359
Clostridiaceae_Clostridium	0.839	0.802	0.955	0.499	0.380	0.210	0.628	0.284
Rikenella	0.175	1.295	0.361	0.300	1.407	0.461	0.572	0.412
Coprococcus	0.421	0.150	0.699	0.747	0.543	0.686	0.123	0.563
Bacteroides	0.450	0.563	0.255	0.257	0.431	0.792	0.259	0.801
Akkermansia	0.448	0.259	0.200	0.430	0.204	0.841	0.710	0.054
Lachnospiraceae_Clostridium	0.288	0.100	0.201	0.236	0.179	0.404	0.791	0.230
Phascolarctobacterium	0.202	0.143	0.177	0.208	0.186	0.881	0.834	0.633
Parabacteroides	0.036	0.131	0.174	0.167	0.125	0.485	0.182	0.423
Blautia	0.215 ^a^	0.106 ^ab^	0.092 ^ab^	0.041 ^b^	0.077	0.051	0.024	0.466
Oxalobacter	0.053 ^ab^	0.031 ^b^	0.110 ^a^	0.069 ^ab^	0.036	0.092	0.015	0.597
Dorea	0.051	0.045	0.014	0.163	0.094	0.143	0.397	0.114
[Ruminococcus]	0.023	0.082	0.097	0.045	0.051	0.891	0.478	0.044
Sutterella	0.037 ^b^	0.030 ^b^	0.101 ^a^	0.056 ^ab^	0.036	0.165	0.022	0.300
Synergistes	0.059	0.032	0.044	0.063	0.066	0.898	0.819	0.505
Butyricimonas	0.031	0.072	0.032	0.048	0.038	0.154	0.562	0.519
Alistipes	0.044	0.030	0.048	0.039	0.045	0.617	0.754	0.915
Lactobacillus	0.004 ^b^	0.135 ^a^	0.006 ^b^	0.033 ^b^	0.055	0.033	0.031	0.025
Others	86.106	84.744	81.849	82.267	3.791	0.806	0.092	0.644

Akebia extract (A), Dandelion extract (D), Standard deviation (SD). Symbol + or − stands for with 0% or 0.5% extract addition in the group. Different lowercase letters indicate a significant difference (*p* < 0.05) between treatments. Abbreviations: A, akebia extract; D, dandelion extract; SD, standard deviation.

## Data Availability

The original contributions presented in this study are included in the article. Further inquiries can be directed to the corresponding author.
